# A novel indirect calorimeter system for measuring gas exchange and determining digestibility, nutrient retention, and net energy in diets fed to group-housed pigs: technical note

**DOI:** 10.5713/ab.25.0397

**Published:** 2025-09-30

**Authors:** Cristhiam J. Munoz Alfonso, Hans H. Stein, Su A Lee

**Affiliations:** 1 Department of Animal Sciences, University of Illinois, Urbana, IL, USA

**Keywords:** Calorimeter, Chamber, Group-housed, Net Energy, Pigs

## Abstract

A novel indirect calorimeter chamber system has been built at the University of Illinois Urbana-Champaign (IL, USA). The unit consists of 6 respiration-metabolism chambers where temperature and humidity are controlled, gas analysis units, a data management unit, a mechanical room, and an air-conditioned feed storage room. The chambers are airtight and each chamber has the capacity to hold more than 1 pig depending on size. Using the system, concentrations of digestible energy, metabolizable energy, and net energy, and digestibility and retention of nutrients can be determined in diets fed to pigs on an *ad libitum* basis. A recovery test was performed using propane combustion. The recovery rate of oxygen ranged from 86.7% to 108.7% with a mean of 99.0% and a coefficient of variation of 6.27%. The recovery rate of carbon dioxide ranged from 100.0% to 101.0% with a mean of 100.6% and a coefficient of variation of 0.29%. In summary, a novel calorimeter unit allows for pigs to be group-housed and given *ad libitum* access to feed and water. This system is believed to be representative of commercial settings, and therefore, net energy can be determined in diets fed to pigs under conditions similar to those used in commercial production.

## INTRODUCTION

In addition to quantifying energy lost in feces, urine, and gas, the net energy (NE) system also accounts for the energy lost in the form of heat and different utilization of metabolizable energy (ME) among nutrients in the body [1–4]. Therefore, it is believed that the NE system is the most accurate system to determine energy in diets for pigs [1,3].

An indirect calorimeter unit using respiration chambers is commonly used to estimate NE of diets for pigs because it allows for the calculation of NE based on the consumption of oxygen (O_2_) and the productions of carbon dioxide (CO_2_) and methane (CH_4_) by animals [2,5]. In most cases, calorimeter chambers allow for only one or two animals to be placed in the chamber at the same time [6–9]. However, under commercial conditions, pigs are kept in groups, and it is likely that group-housed pigs have different energy expenditures than individually housed pigs, which may affect estimated values for NE. It is, therefore, possible that NE values that accurately reflect what is obtained in commercially housed pigs need to be determined in group-housed pigs. Likewise, because commercial pigs usually are allowed *ad libitum* access to feed, and because the level of feed intake may affect the digestibility of nutrients and energy and post-absorptive energy metabolism [10], it is possible that NE values that are obtained in pigs allowed *ad libitum* intake of feed are more representative of commercial pigs than if pigs are restricted in their feed intake. Therefore, a novel indirect calorimeter system has been constructed to determine nutrient and energy digestibility and ME and NE of diets fed to group-housed pigs that are allowed *ad libitum* access to diets. The hypothesis of this work was that the newly developed indirect calorimeter system provides consistent gas exchange measurements across chambers, which enables accurate estimation of NE in diets fed to group-housed pigs.

## MATERIALS AND METHODS

### Unit descriptions

**Figure 1:**
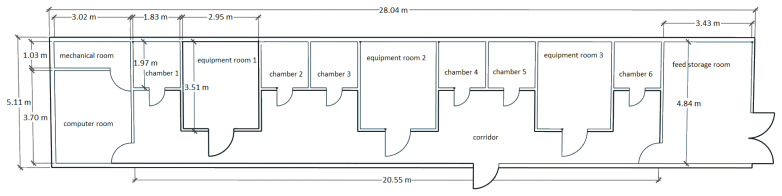
Schematic layout of the indirect calorimeter system at the University of Illinois Urbana-Champaign (IL, USA).

The respiration-metabolism chamber unit is located at the Swine Research Center at the University of Illinois Urbana-Champaign, IL, USA (Figure 1). The outside of the chamber unit measures 28.04 m×5.11 m. The unit is a wood-framed construction that is placed on a steel chassis, with oriented strand board walls, a wood truss roof, and a plywood floor. All surfaces on the inside are coated with sprayed-on plastic for watertightness. This unit consists of 6 respiration-metabolism chambers where temperature and humidity are controlled, 3 gas analysis units, a data management unit (i.e., computer room), a mechanical room where utility equipment for maintenance is located, and an air-conditioned feed storage room. The chambers are airtight, and each chamber has the capacity to hold 2 to 10 pigs depending on size and the minimum floor space for pigs recommended by the Ag Guide 4^th^ ed. ([11]; Table 1).

### Respiration-metabolism chambers

**Figure 2:**
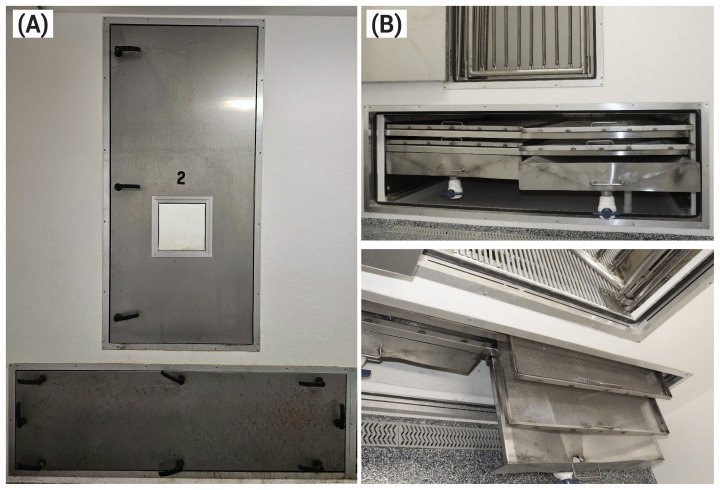
Photos of (A) front side of a respiration-metabolism chamber (airtight) and (B) the secondary section with 4 screens and 2 pans for fecal and urine collections.

Each chamber is composed of a main section for animals and a secondary section that allows for separate collection of feces and urine (Figure 2). The inner dimensions are 1.83 m×1.97 m with a height of 1.8 m and a volume of 6.5 m^3^. The door of the main chamber is airtight, has a gasket surface, is side-hinged, and contains 3 rubber-metal handles for closing. The ceiling and walls in the main section are constructed from a wood-coated frame with sprayed-on plastic. The floors consist of galvanized steel slotted panels and the contact surface is formed by evenly spaced triangular bars. The floor is self-supporting at the ends by means of an engineered steel under-slat truss system. The contact surface is deformed to improve hoof traction. The support truss is designed for the maximum animal load and module length. An air supply duct and diffuser are located in the ceiling of each chamber, and an air outlet is located on the side wall of each chamber. The chamber contains a stainless steel wet-dry feeder with the capacity of 30 kg (Thorp Equipment). An auxiliary drinker is also available in each chamber to ensure free access to water.

The inner dimensions of the secondary section are 1.83 m×1.97 m with a height of 0.86 m and a volume of 3.1 m^3^. The secondary chamber door is airtight, sealed by a gasket surface, and secured with eight rubber-coated metal handles for complete closure. The secondary section has 4 flat stainless steel wire mesh screens with openings of 1,190 microns for feces collection. The measurements of these screens are 0.91 m×1.97 m. The screens are placed in parallel and in two rows with a 10 cm separation between screens to avoid sample loss during collection. During collection periods, both the main and secondary sections of the chambers are open. To collect feces, the screens in the upper row in the secondary section are pulled out from their mounting, and the feces on the screens are collected as well as feces that stay in the main section. After cleaning the upper screens and placing them back under the slatted floor, the lower screen row is pulled out to collect the fecal material that may have been voided while the upper screen row was pulled out for collection of feces. The 2 urine pans are placed below the screens and have a total capacity of 100 L. The pans are equipped with a manual valve, which allows for the collection of urine from the access corridor. To avoid nitrogen loss from the urine, 200 mL of 6 *N* HCl are placed in the urine pans each day after collection of urine.

### Equipment rooms

There are 3 equipment rooms that each has a volume of 27.64 m^3^, with inner dimensions of 2.95 m×3.51 m×2.67 m. The equipment placed in these rooms includes systems to control temperature and relative humidity within the chambers, equipment to supply fresh air to the chambers, and equipment to analyze collected air samples for O_2_, CO_2_, and CH_4_. Additional gas analyzers can be installed if needed. The equipment in each room serves two calorimeter chambers that are located on either side of each equipment room, which ensures that the gas lines from the chambers to the gas analyzers are very short to reduce the risk of loss of gases.

#### Temperature and humidity control system

**Figure 3:**
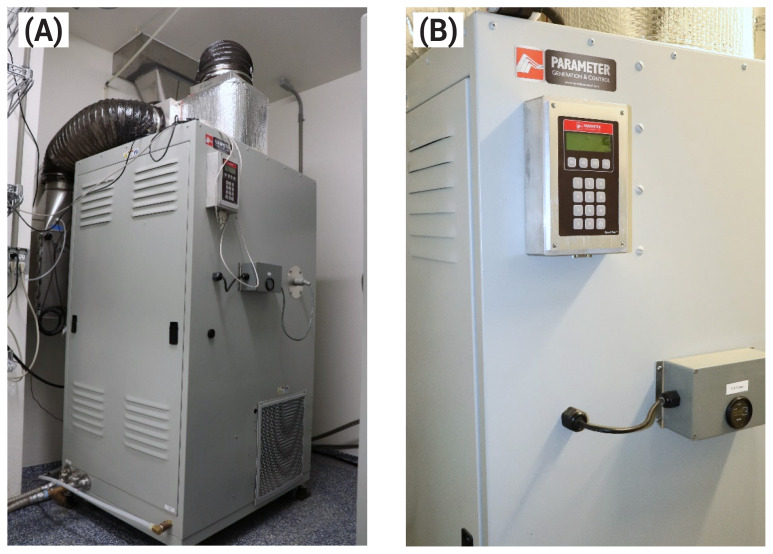
Photos of the Parameter Generation and Control (PGC) unit (A); and a detailed view of the control panel and display of the PGC unit (B). Temperature and humidity within each chamber can be adjusted and monitored using the control panel of the PGC unit.

The temperature and humidity in each chamber are controlled by the Parameter Generation and Control (PGC) unit (Model 9241-2220-B1D0000; Parameter). There are a total of 6 PGC units, which are located in the 3 equipment rooms, with 2 PGC units per room (Figure 3). The PGC units control the temperature with an accuracy of ±0.1°C and relative humidity is maintained with an accuracy of ±0.5%. This level of precision is ensured by the use of the dew point control system, which operates by manipulating the air temperature going through the PGC unit and the temperature of the water spray that saturates the air with moisture and thus, controls the humidity and temperature in the chamber [12]. Because temperature and humidity in each chamber are individually controlled by each PGC unit, different temperature and humidity conditions can be maintained in each chamber. This allows simulation of weather conditions with various temperature and humidity as long as the dew point of the gas flowing through the sample lines and the gas analysis stack does not exceed limits determined using a psychrometric chart.

The air blower in each PGC unit has a capacity of 700 to 1,100 m^3^ of air per hour. The unit consists of 316-grade stainless steel, with electricity requirements of 208/230V, 3-Phase, 60 Hz, 22.5 full-load amperes, and 14.1 rated load amperes. The weight of each PGC unit is 522 kg and dimensions are 0.81 m×1.02 m×1.63 m. The rated maximum heat of rejection for each unit is approximately 7,300 watts/h. The PGC unit provides instant readings of temperature and humidity in the chamber and allows programming for automatic cycles, set points, and tuning parameters. All information can be continuously monitored on the master computer connected to the PGC unit. The equipment also contains an alarm system that is activated if the temperature or the humidity in the chamber deviates from the set allowances.

#### Fresh air supply system and air exchange

**Figure 4:**
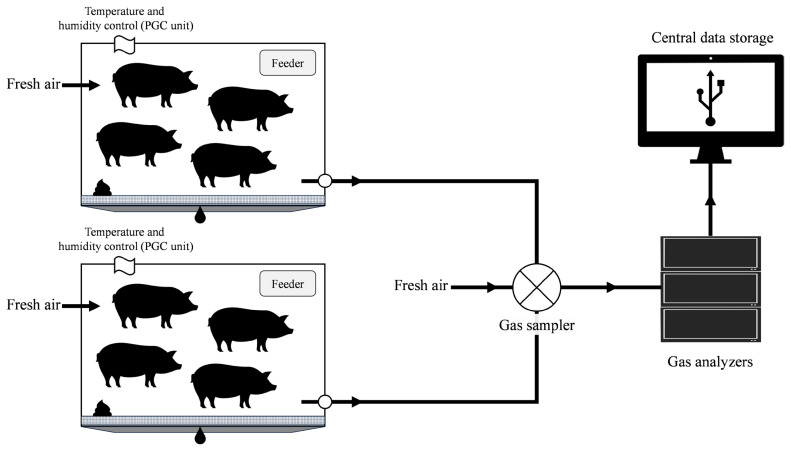
A schematic diagram of the airflow for two chambers. Each chamber is equipped with its own Parameter Generation and Control (PGC) unit and both chambers share a gas sampler and gas analyzers located in an equipment room. Fresh air is supplied to each chamber; air samples from the two chambers and a baseline are collected by the gas sampler located in the equipment room; and the gases from 3 lines (i.e., fresh air, chamber 1, and chamber 2) are analyzed by the gas analyzers located in the equipment room. The resulting gas data are then transmitted to the central data storage located in the computer room. The indirect calorimeter system consists of three identical chamber pairs as described in this figure.

The fresh air supply system introduces fresh air to each chamber to provide clean air to pigs and to the gas analyzers to generate the baseline data (Figure 4). There is one air supply system for each chamber. These systems consist of a centrifugal inline fan, which has a maximum rated airflow of 293 m^3^/h, and is constructed of galvanized sheet metal (Fantech). The AccuValve (Accutrol LLC) is also part of the system with a length of 56 cm and a diameter of 15 cm. The AccuValve divides the airflow into 2 equal flows, which pass through the airflow sensor; a measure of this airflow is sent to the digital controller where the airflow set point is calibrated. The controller modulates the blades inside the AccuValve to achieve the airflow determined by the set point and moderates the airflow to be sucked into the calorimeter chamber by the fan. The AccuValve has an accuracy of +/− 5% and a maximum airflow rate of 509 m^3^/h and is equipped with 90 cm of a 15 cm diameter PVC pipe on each side to allow maximum accuracy according to manufacturer recommendations.

The air exchange in the chamber is set by the AccuValve and chamber pressure is regulated by a manual rotary plate valve located in the exhaust duct, which allows chamber air to vent to the outside of the building. The manual valve is set to maintain a small positive pressure in the chambers and to avoid entrance of external air to the chamber. A manometer (Dwyer) with an accuracy of 3% is attached to the exhaust pipe, to maintain the chamber pressure at 174.19 kPal at all times.

#### Gas analyzers

Two chambers share one gas analysis system (Classic Line, Sable System International) and this system is equipped with 2 pumps, a multiplexer, a sub-sampler, a humidity sensor, and 3 different gas analyzers (i.e., O_2_, CO_2_, and CH_4_). The air from the chamber return duct is collected and transported to the multiplexer by the pumps. The fresh air is also collected and transported to the multiplexer to generate the baseline data. The multiplexer is programmed to select one of the 3 lines of airflow at a time and direct the airflow to the sub-sampler. The sub-sampler pulls 250 mL±10% per minute of air from the chamber through the gas analyzers. Before the air stream enters the gas analyzers, it passes through the humidity sensor, which detects water molecules by infrared spectroscopy. The sensor generates relative humidity values that are used as a correction factor for gas analyses. The air subsample first enters the CO_2_ analyzer, then the CH_4_ analyzer, and finally the O_2_ analyzer. The gas analyzers provide readings in percentage units. The resolution of the analyzer is 0.0001, but this resolution can vary depending on gas concentration.

#### Dry cooling system

The dry cooling system (Vertiv Tm) is used to provide heat transfer between the PGC unit’s refrigeration condensers and the atmosphere outside the building. The system contains 2 exhaust fans that exhaust the heat that is transferred from the PGC’s metal surface that contains the refrigerant to the propylene glycol-filled finned tubes [13].

The dry cooling system consists of an outside fan-cooled radiator, an electric pump that distributes the fluid cooler through a manual balancing valve, which produces a pressure difference across the supply and return sides of the glycol loop, and another pump for backup. The fan-cooled radiator is located in an aluminum cabinet outside the calorimeter unit and its dimension is 1.1 m×2.3 m×1.1 m. The dry cooler glycol loop includes a 30-L expansion tank. Its dimensions are 0.76 m×0.82 m×0.48 m and it is placed in an aluminum drip-proof case. The dry cooler system can handle 21,370 m^3^ of air per hour. The fluid flow rate is 75 L/min, which allows the system to remove 42,202 watts of heat from the system per hour.

### Determination of energy concentrations in feeds fed to pigs housed in indirect calorimeter chambers

Determining concentrations of digestible energy (DE), ME, and NE, and apparent total tract digestibility of nutrients in diets fed to pigs in the chambers as well as retention of nutrients follow established principles for conducting digestibility experiments [14]. Pigs in each chamber are allowed *ad libitum* access to feed and water from the feeder and nipple drinker. Diets are fed for 13 d, where the initial 7 d are considered the adaptation period to the diet. Feeders are checked twice daily. Daily feed allotments and residual feed at the end of collection period are recorded to calculate feed consumption. Fecal and urine samples are quantitatively collected from d 8 to d 13 and O_2_ consumption and CO_2_ and CH_4_ productions are also measured during this time. During the collection period and to avoid nitrogen loss in the urine, 100 mL of 6 *N* HCl is added to each urine pan every day to have a total of 200 mL of 6 *N* HCl in each chamber. Fecal samples and 5% of the collected urine are stored at −20°C immediately after collection. Following collection, all collected feces are dried (Heratherm OMH750; Thermo Fisher Scientific) and ground using a hammermill (MM4; Schutte Buffalo). A subsample is then collected for chemical analysis and the apparent total tract digestibility of energy and nutrient is calculated. Urine samples are also analyzed for energy [15] and nutrients, and nutrient retention as well as ME are calculated.

Fasting heat production can also be estimated in this system. Pigs are deprived of feed for 36 to 48 h. The initial 24 or 36 h of the fasting period is assumed to be the time in which the animals digest and metabolize the remaining feed in the intestinal tract to produce energy, whereas gas exchanges are measured, and urine is collected during the following 12 h, which is assumed to be the period in which the animals mobilize endogenous nutrients to produce energy. It is also possible to calculate fasting heat production by estimates of 137 kcal/kg body weight^0.60^ [16], 179 kcal/kg body weight^0.60^ [17], or 167 kcal/kg body weight^0.60^ [18].

Chambers are opened every day to check feeders and add feed as needed, and for collection of fecal and urine materials from the chambers. Data from the gas analyzers obtained during the periods when chambers are open and until they reach the condition set by the temperature and humidity control unit are disregarded in the calculation of heat production. Total and fasting heat productions per hour (kcal/h) are calculated using the following equation [19]:


(1) $$\text{Heat production }(\text{kcal}/\text{h})=(3.866\times {\text{O}}_{2})+(1.2\times {\text{CO}}_{2})-(0.518\times {\text{CH}}_{4})-(1.431\times \text{urine N}),$$

where O_2_ consumption and CO_2_ and CH_4_ productions are expressed in L/h and urine N is in g/h per chamber. The heat production per hour is then multiplied by 24 to calculate daily heat production. Heat increment is calculated by subtracting fasting heat production from total heat production and NE is then calculated using the following equation (modified from NRC [4]):


(2) NE (kcal/kg)=ME-(Total heat production-Fasting heat production)/Feed intake,

where ME is expressed in kcal/kg, which is calculated by subtracting energy in feces and urine; total heat production and fasting heat production are in kcal/d for each chamber; and feed intake is kg/d for each chamber.

### Recovery test using propane combustion

To check the accuracy of gas exchange measurements from each chamber, a recovery test for O_2_ and CO_2_ is conducted using propane gas combustion. This test is conducted for each of the 6 indirect calorimeter chambers when no pigs are housed in the chamber. Theoretically, propane (molecular formula: C_3_H_8_) reacts with O_2_ and produces CO_2_ and water when combusted as follows:


(3) $${\text{C}}_{3}{\text{H}}_{8}+5{\text{O}}_{2}\to 3{\text{CO}}_{2}+4{\text{H}}_{2}\text{O},$$

which indicates that for each mole of propane burned, 5 moles of O_2_ are consumed, and 3 moles of CO_2_ are produced.

The baseline concentrations of O_2_ and CO_2_ are determined from the fresh air going into the chambers. Propane gas from a portable fuel cylinder containing approximately 100% propane (453 g propane per cylinder; Coleman) is released at a rate of 160 to 220 g/hour and combusted within each chamber. The amount of propane combusted is determined by weighing the cylinder before and after combustion and any surface moisture is removed prior to weighing. This difference is then used to calculate the expected amount of O_2_ consumption and CO_2_ production based on the stoichiometry of propane combustion.

Concentrations of O_2_ and CO_2_ are recorded for an hour. The recovery rate (%) is calculated by dividing the measured amount of O_2_ consumption or CO_2_ production by the expected amount of O_2_ consumption or CO_2_ production and multiplying by 100.

## RESULTS AND DISCUSSION

### Determination of energy concentrations in feeds fed to pigs housed in indirect calorimeter chambers

Using the new system, concentrations of DE, ME, and NE, apparent total tract digestibility of nutrients, and retention of nitrogen in diets fed to group-housed pigs have been successfully determined [20,21]. In these experiments, total heat production, fasting heat production, concentrations of NE, and NE-to-ME ratio in diets were greater in group-housed pigs compared with previously reported values from individually housed pigs [9,17]. The greater total heat production is likely due to greater feed intake of group-housed pigs fed *ad libitum* compared with individually housed pigs fed restrictively [21], which may reduce NE in diets. However, fasting heat production, which represents the greatest part of maintenance [4], was also greater in group-housed pigs. This is likely due to increased maintenance energy needs associated with social interactions and activity and gastrointestinal tract and liver mass [4,22], which in turn contributed to the increased NE in diets fed to group-housed pigs.

### Recovery test using propane combustion

The recovery rate of O_2_ ranged from 86.7% to 108.7% with a mean of 99.0% and a coefficient of variation of 6.27% (Table 2). The recovery rate of CO_2_ ranged from 100.0 to 101.0% with a mean of 100.6% and a coefficient of variation of 0.29%. The observation that the recovery rate of O_2_ and CO_2_ was close to 100% indicated that the indirect calorimeter chamber system provides accurate and precise measurements for O_2_ and CO_2_. The recovery test is conducted in each chamber before each experiment and after gas analyzers are calibrated.

If the recovery is not close to 100%, the test is repeated using a new bottle of propane. In some cases, incomplete combustion may occur because of poor supply of propane from a nearly empty or defective bottle. Therefore, it is recommended to use a full bottle to ensure propane is consistently supplied. Additionally, the airflow rate should be checked. It is also recommended to inspect leaks in the sampling lines and in the duct connecting the chamber to the equipment room.

## CONCLUSION

A novel indirect calorimeter system for measuring gas exchange and determining apparent total tract digestibility of nutrients and energy and concentrations of DE, ME, and NE in diets fed to group-housed pigs has been constructed at the University of Illinois. In this system, environmental conditions in each chamber are controlled to maintain temperature and humidity in the comfort zone for pigs and thereby limit energy losses in the form of heat used for thermoregulation. Each chamber has the capacity to hold more than 1 pig depending on size and pigs are allowed *ad libitum* access to feed and water, which is more representative of commercial settings than if pigs are housed individually and fed restricted amount of feed. Regular recovery tests are needed to ensure the accuracy of gas analyzers because gas exchange measurements are critical for calculating heat production by pigs.

## Figures and Tables

**Table 1:** Recommended capacity per calorimeter chamber

Body weight of pigs (kg)	Number of pigs in a chamber
11 to 20	10
21 to 40	6
41 to 60	6
61 to 80	4
81 to 100	4
101 to 130	4
Adult pigs in groups (e.g., sows)	2

**Table 2:** Oxygen (O_2_) and carbon dioxide (CO_2_) recovery test using propane in six indirect calorimeter chambers

Item (%)	O2 recovery^1)^	CO2 recovery^1)^
Chamber		
1	99.6	100.6
2	98.0	100.6
3	98.3	100.7
4	99.5	100.6
5	100.5	100.6
6	97.4	100.3
Statistical metrics		
Mean	99.0	100.6
Maximum	108.7	101.0
Minimum	86.7	100.0
Standard deviation	6.20	0.30
Coefficient of variation (%)	6.27	0.29

Data were calculated using 4 or 5 observations per chamber.

1) Oxygen recovery rates (%) were calculated by dividing the measured amount of O_2_ consumption by the expected amount and multiplying by 100; CO_2_ recovery rates (%) were calculated by dividing the measured amount of CO_2_ production by the expected amount and multiplying by 100.
